# Cannabidiol Intervention for Muscular Tension, Pain, and Sleep Bruxism Intensity—A Randomized, Double-Blind Clinical Trial

**DOI:** 10.3390/jcm13051417

**Published:** 2024-02-29

**Authors:** Karolina Walczyńska-Dragon, Anna Kurek-Górecka, Wojciech Niemczyk, Zuzanna Nowak, Stefan Baron, Paweł Olczyk, Aleksandra Nitecka-Buchta, Wojciech M. Kempa

**Affiliations:** 1 Department of Temporomandibular Disorders, Medical University of Silesia in Katowice, Traugutta Sq. 2, 41-800 Zabrze, Poland; s81190@365.sum.edu.pl (W.N.); zuzanna.nowak@sum.edu.pl (Z.N.); sbaron@sum.edu.pl (S.B.); aleksandra.nitecka@sum.edu.pl (A.N.-B.); 2 Department of Community Pharmacy, Medical University of Silesia in Katowice, Kasztanowa 3, 41-205 Sosnowiec, Poland; akurekgorecka@sum.edu.pl (A.K.-G.); polczyk@sum.edu.pl (P.O.); 3 Department of Mathematics Applications and Methods for Artificial Intelligence, Faculty of Applied Mathematics, Silesian University of Technology, Akademicka 2A, 44-100 Gliwice, Poland; wojciech.kempa@polsl.pl

**Keywords:** temporomandibular joint disorders, cannabidiol, facial pain, bruxism

## Abstract

**Background**: Temporomandibular disorders (TMDs) are the most prevalent non-dental pain issues in the maxillofacial region. Despite advancements, diagnosing and managing TMDs continues to pose challenges. This study aimed to assess the efficacy of cannabidiol (CBD) formulations, with different concentrations, in patients experiencing sleep bruxism and muscle-related TMDs, with a particular emphasis on their myorelaxant, pain-relieving, and bruxism-reducing properties. **Methods**: The Research Diagnostic Criteria for Temporomandibular Disorders (RDC/TMDs) was utilized as the diagnostic framework. Sixty patients completed the study, which followed a parallel-group, three-arm, randomized, double-blind clinical trial design, with a 1:1:1 allocation ratio across three groups: 1a, 1b, and 2. Groups 1a and 1b received CBD formulations at concentrations of 10% and 5%, respectively, while Group 2 received a placebo formulation. The trial consisted of four main visits, namely screening, baseline, first follow-up after 14 days, and second follow-up after 30 days, during which surface electromyography (sEMG), the visual analogue scale (VAS) for pain assessment, and Bruxoff examinations were conducted. **Results**: The reduction in pain, as measured by the visual analogue scale (VAS), among patients using the 10% CBD formulation was 57.4% (*p* < 0.05), accompanied by a decrease in sEMG activity by 42.1% (*p* < 0.05). Conversely, individuals using the 5% CBD formulation experienced a 40.8% (*p* < 0.05) decrease in pain. Regarding the decrease in the sleep bruxism index, users of the 10% CBD formulation saw the highest reduction of 51% (*p* < 0.05). These findings underscore the efficacy of the proposed treatment in both experimental groups, with a notable advantage observed in Group 1a. Conversely, the outcomes of the selected variables for the control group did not exhibit significant differences throughout the study. **Conclusions**: The intraoral use of CBD formulations in patients with TMDs have proven to be a successful treatment for reducing pain, muscle tension, and bruxing activity in individuals with sleep bruxism and muscle-related TMDs. Specifically, a concentration of 10% CBD has demonstrated superior results compared to 5% CBD.

## 1. Introduction

The term “temporomandibular disorders” (TMDs) collectively refers to a group of musculoskeletal conditions affecting the temporomandibular region [1]. Current guidelines delineate 12 common TMDs, encompassing conditions such as arthralgia, myalgia, local myalgia, myofascial pain, myofascial pain with referral, four-disc displacement disorders, degenerative joint disease, subluxation, and headache attributed to TMDs [2]. TMD-associated pain is classified as either acute or chronic, with patients often experiencing symptoms such as muscle pain, restricted jaw movements, otologic symptoms, heightened tooth pain or sensitivity, cephalalgia, temporomandibular joint-area pain, periorbital pain, and constrained cervical movements. These clinical manifestations can significantly impact an individual’s sleep patterns, quality of life, and psychological well-being [3,4]. Myalgia may present as referred pain, extending to the cranio-cervical region, resulting in concomitant symptoms, such as tinnitus, otalgia, ocular discomfort, migraines, and distinctive non-odontogenic dental pain [5,6]. Recurrent muscle-related temporomandibular disorders (TMDs) often arise due to hyperactivity and excessive use of masticatory muscles, often triggered by bruxism, which is recognized as a contributing factor to a range of dental pathologies, including periodontal complications, dental and radicular fractures, prosthetic malfunction, and tooth wear [7]. Bruxism has been categorized into two forms based on circadian rhythms: sleep bruxism (SB) and awake bruxism (AB) [8]. Sleep bruxism, as defined by Lobbezoo et al. (2018), entails masticatory muscle activity (MMA) during sleep, characterized by rhythmic (phasic) or non-rhythmic (tonic) patterns. Importantly, it is not classified as a movement or sleep disorder in otherwise healthy individuals. In contrast, awake bruxism involves masticatory muscle activity during wakefulness, typically marked by repetitive or sustained dental contact and/or mandibular bracing or thrusting. Similar to SB, AB is not categorized as a movement disorder in individuals without underlying health issues [9]. The etiology of bruxism largely involves biological, psychological, and social factors, necessitating a multifaceted treatment approach [10]. Over time, various therapeutic modalities have been suggested, with some becoming outdated, while others have gained traction. However, due to the diverse range of symptoms associated with TMDs, finding a universal remedy remains challenging [6]. First-line therapeutic approaches typically include occlusal splint therapy, physical therapy, patient education, and behavioral modifications aimed at addressing parafunctional habits [11,12,13]. Minimally invasive interventions, such as intramuscular needling, and the application of substances like botulinum toxin, collagen, platelet-rich plasma (PRP), or anesthetics, may serve as secondary treatment options [14]. Therapy may be augmented by pharmacotherapy and adjunctive measures, including appropriate supplementation, dietary adjustments, and sleep pattern optimization [10].

Recent attention has been directed towards cannabidiol (CBD) as a potential adjunct in alleviating orofacial myofascial pain [15]. CBD is considered to regulate many physiological processes, such as pain sensation or inflammation. Despite limitations, significant evidence is described on the therapeutic effects of CBD, including anticonvulsant, antipsychotic, chronic pain relief, muscle-relaxing, anxiolytic, neuroprotective, and sleep-promoting effects [16,17,18,19,20,21].

In addition to acting on cannabinoid CB1/CB2 receptors, CBD may reduce pain through its interaction with the putative non-CB1/CB2 cannabinoid G protein-coupled receptor (GPCR) 55 or GPCR 18 (GPR18), also known as the N-arachidonoyl glycine (NAGly) receptor, and other well-known GPCRs, such as opioid or serotonin (5-HT) receptors, which contribute to the effectiveness of low-dose CBD for reducing stress, anxiety, and pain. Moreover, CBD antagonizes alpha-1 adrenergic and µ-opioid receptors, which may contribute to the somnogenic and anti-inflammatory effects of the substance. Therefore, CBD is responsible for inhibiting the synaptosomal uptake of noradrenaline, dopamine, serotonin, and gamma-aminobutyric acid, and the uptake of anandamide in cells. It also inhibits sodium and calcium channels, thereby dampening nerve excitability, which may contribute to reducing pain hypersensitivity and seizures [22,23,24]. Researchers are increasingly exploring strategies for administering CBD to individuals suffering from temporomandibular disorders characterized by muscle hyperactivity or inflammation of the temporomandibular joint. Grossman et al. concluded, based on a systematic review, that there is a considerable amount of high-quality evidence supporting the use of cannabis-based products for treating chronic neuropathic and nociceptive pain. However, they noted that evidence specifically related to orofacial symptoms is very limited [15]. The authors of the following randomized trial aimed to evaluate the effects of intraoral CBD gel application on the reduction of pain, the bruxism index (BRK), and muscle activity in patients suffering from muscle-related TMDs.

## 2. Materials and Methods

This study employed a parallel-group, three-arm, randomized, double-blind clinical trial, with a 1:1:1 allocation ratio. Patients were recruited from those referred to the Department of Temporomandibular Disorders at the Medical University of Silesia, Poland.

### 2.1. Study Participants

Patients were selected from a group of 79 patients attending the Department of Temporomandibular Disorders, Medical University of Silesia, Poland. After considering the inclusion and exclusion criteria, qualified patients (*n* = 66) were randomly assigned to three groups by allowing them to select a number (1a, 1b, or 2). The inclusion and exclusion criteria for patient qualification are outlined in Table 1. Of the 66 patients included in the trial, 60 completed the study, with 20 participants in each group. We used Slovin’s formula to compute the sample size. Indeed, assuming the population size (the total number of patients) *N* = 150, and taking into account the level of error tolerance *e* = 0.1 (10%), we get:

$$n=\frac{N}{1+N\cdot e^{2}}=60$$

This study was approved by the Bioethical Committee at the Medical University of Silesia (number PCN/0022/KB1/66/II/20/21, consent obtained on 20 April 2021) and was prospectively registered at ClinicalTrials.gov (NCT05562635) (accessed on 31 August 2022). This research adhered to the principles outlined in the Declaration of Helsinki and the International Conference on Harmonization guidelines for Good Clinical Practice. Patients were provided with both verbal and detailed written explanations of the trial and provided consent to participate in the study.

### 2.2. Study Protocol

The research adhered to the Consolidated Standards of Reporting Trials (CONSORT) statement [25] and was conducted from 1 January 2023 to 30 May 2023, within the Department of Temporomandibular Disorders at the Medical University of Silesia.

During visits, patients were examined by experienced dentists (KWD, ANB). The clinical assessment followed the RDCTMDs criteria. Patients were included if the examination resulted in a positive outcome in relation to the criteria: II.1.A. 1, 2, and 3 [26].

Randomization was performed by ANB, a dentist not involved in the follow-up visits. Patients were randomly assigned to one of three groups, Experimental 1a, Experimental 1b, or Control 2, by selecting containers with CBD gel or a placebo, prepared by AKG. Formulations 1a and 1b contained a viscous cream-colored preparation with a slightly bitter taste. The placebo formulation contained a viscous cream-colored preparation that was nearly tasteless. Each formulation was packaged in Uno Dose containers and taped in a manner to prevent patients from identifying the applied formulation. Each container was tightly sealed and opaque, ensuring that the patients could not discern the contents before selection. Patients in the Experimental 1a group received the 10% CBD formulation, those in Experimental 1b received the 5% CBD formulation, and those in the Control 2 group received the placebo without CBD (polymer gel containing only Celugel and oils). The study was conducted as a double-blind trial, ensuring that neither the doctor nor the patient knew to which group the patient was assigned. 

The study comprised four scheduled appointments:

1. Screening visit: examination and inclusion assessment, provision of a Bruxoff device for home examination of sleep bruxism. The initial qualification test included a dental examination, functional evaluation of the stomatognathic system, subjective medical history review, and allergy assessment for the CBD formulation. Sleep bruxism intensity was determined by the bruxism index (BRK), which measures the number of bruxism episodes per hour.
2. Baseline visit: participants underwent a surface electromyography (sEMG) test (sEMG I) and reported their pain levels using the visual analogue scale (VAS I). Additionally, patients were randomized into their respective groups.
3. First follow-up visit: occurred after 14 days of application and included a follow-up sEMG assessment (sEMG II) and VAS assessment (VAS II).
4. Second follow-up visit: scheduled after 30 days of application, this visit involved a follow-up sEMG assessment (sEMG III), VAS assessment (VAS III), and Bruxoff examination.

### 2.3. Preparation of CBD Formulation, Application of CBD or Placebo Formulation

An experimental gel (for Group 1a and 1b) containing CBD was formulated by mixing 100% CBD isolate in powder form with hydrogel, based on hydroxyethyl cellulose (Celugel, Actifarm, Permit No 30050), to achieve polymeric gels with CBD concentrations: 5% and 10%, respectively. Additionally, paraffin oil was employed as a levitating liquid. A transparent hydrophilic gel delivered by Actifarm, Poland, served as the fat-free base medium. It is formed from a semi-synthetic, organic macromolecular colloid, hydroxyethyl cellulose, using a solvation process. It contains hydroxyethylcellulose 10,000, glycerol 85%, aqua purificata, sorbic acid, and potassium sorbate. Hydrogel containing CBD was prepared in an Eprus^®^ U500 automatic recipe mixer. Additionally, paraffin oil was utilized to facilitate the grinding of the CBD powder. CBD was mixed manually with paraffin oil in a mortar to micronize the substance. After grinding the CBD in a mortar with paraffin oil for 2 min at room temperature, the CBD was blended with the hydrogel for 8 min at a speed of 1960 RPM. The CBD gel was placed in special containers (Uno Dose, Eprus PN-EN ISO 15378:2018) [27], which ensured the use of the same, measured dose of gel by each patient every day. The gel was sealed in a container and the patients were instructed to turn the knob at the bottom of the container once to obtain 1 dose of the gel. The dosing knob advances the elevator to dispense the dosage, alerting the user by sound, sight, and feel. One turn of the knob delivers 0.2 g of the gel, as confirmed by the laboratory scale measurements. In the group receiving 10% CBD gel, the patients used 20 mg (0.02 g) CBD per side, i.e., 40 mg CBD daily, before bedtime. Similarly, the patients using 5% CBD gel applied 20 mg (0.02 g) CBD daily. The use of Uno Dose containers helped reduce the risk of errors. The patients were instructed to apply the gel to both the right and left masseter muscle intraorally, administering one full dose per side, before going to sleep. They were also instructed not to drink any beverages after applying the gel. The control group received the polymer gel without CBD. 

### 2.4. Bruxoff Measurements—Bruxism Assessment

Bruxoff (Bruxoff^®^, Spes Medica, Genova, Italy) is a portable device providing combined sEMG and electrocardiography (ECG) measurements [28]. The device features three channels, which capture electromyographic signals bilaterally from the masseter muscles and the heart to monitor its frequency rate. These signals were sampled at 800 Hz, with an 8-bit resolution, and the data was stored on a MicroSD card in binary format. The surface EMG channels were filtered between 10 and 400 Hz, with the gain set to 4300 Hz. The ECG channel was filtered between 15 and 160 Hz, with the gain set at 700 Hz. Bruxoff examinations were performed during sleep by the patients themselves, following the clinicians instructions. Patients were instructed on how to apply disposable surface electrodes (Spes Medica, Genova, Italy) above the masseter muscles bilaterally and on the thorax. The dentist (KWD) instructed the patients on how to properly place the electrodes. Heart frequency was detected using a disposable bipolar electrode placed on the left side of the thorax, just below the pectoral muscles, approximately 5–10 cm below the sternum. Before commencing the recordings, each patient was instructed to calibrate the device, by performing three series of maximum voluntary clenching (MVC), each lasting for 2 s with a 5 s break in between. The highest MVC value obtained was used to normalize the EMG values as a percentage of the MVC. Automatic scoring of the Bruxoff recordings was performed by the Bruxmeter software v.2.0.2.7 (OT Biolettonica, Torino, Italy). Bruxoff was employed to measure various data parameters, including the bruxism index, mean heart rate, number of masseter muscle contractions (tonic, phasic, and mixed), and examination length (the duration between device activation and deactivation, excluding specific sleep durations). The data were collected, recorded, and evaluated by the same trained dentist (KWD). Bruxism events were categorized as either tonic (characterized by sustained EMG bursts lasting more than 2 s), phasic (consisting of 3 or more rhythmic EMG bursts lasting between 0.25 and 2 s each), or mixed (involving both sustained and rhythmic patterns).

### 2.5. sEMG Measurements—Muscle Tension Assessment

The four-channel Neurobit Optima 4.0 (Neurobit Systems, Gdynia, Poland) system was used to measure the masseter muscle tension (μV), both at rest and during maximal contraction on both sides. The placement of the electrodes was determined based on the anatomical landmarks identified during the baseline visit and consistently located using paper templates. The electrodes were positioned near the upper origin, under the zygomatic arch, and on the angle of the mandible, with a minimum distance of 10 mm between each electrode. To ensure precise electrode positioning, the patients were instructed to execute an isometric contraction of the masseter muscles. The placement of the electrodes and skin preparation followed the SENIAM guidelines: any facial hair was shaved if required, and the skin was cleaned with alcohol (Surface ElectroMyoGraphy for the Non-Invasive Assessment of Muscles, www.seniam.org (accessed on 25 April 2021). Four Ag/AgCl adhesive electrodes, each with a diameter of 30 mm (Sorimex, Toruń, Poland), were bilaterally applied, directly over the masseter muscles. Only electrodes with an adhesive conductive gel overlying the electrode surface were utilized. Reference electrodes were positioned on the neck. Patients maintained a quiet and relaxed environment, seated upright with their feet planted on the floor, and their gaze directed forward, ensuring the occlusal plane remained parallel to the floor. The surface electromyography (sEMG) signal was then amplified and digitized. Initially, resting position (RP) sEMG signals were recorded. To achieve this, the mandible was allowed to rest without any contact between the teeth, with its position maintained solely by the force of gravity and the viscoelasticity of the stomatognathic system tissues. Patients were instructed to swallow their saliva a few times and to refrain from clenching their teeth afterwards. Subsequently, the sEMG signals were recorded during a maximal voluntary isometric contraction (MVIC), established as the reference value. Each test was repeated three times for each muscle on each side, with at least 1 min of rest between the tests. The sEMG tests were conducted by the dentist (ZN) on day 0, day 14, and day 30. The clinician was not aware of the patient’s group. The average sEMG values (μV) for the masticatory muscles during the mandibular RP and MVIC were recorded using a Bioexplorer Neurobit (Neurobit Optima for sEMG, Neurobit Systems, Gdynia, Poland) for the right (EMG-R) and left (EMG-L) masseter. These values were saved, normalized, and analyzed. The planning and execution of the sEMG recordings adhered to the Standards of Instrumentation of EMG [29].

### 2.6. Pain Assessment

During all visits, patients reported their level of maxillofacial pain using the VAS, which was included as a part of the questionnaire. The VAS ranged from 0 to 10, where 0 indicated no pain and 10 indicated the worst possible pain.

### 2.7. Statistical Analysis

Descriptive statistics for the variables under consideration were provided, followed by a normality test using the Shapiro–Wilk test. Moreover, results from the ANOVA analysis and post hoc tests for selected variables were presented. Distribution compatibility tests for specific pairs of independent variables were performed, including the Wald–Wolfowitz test, Kolmogorov–Smirnov test, and Mann–Whitney U test. To compare probability distributions across all three groups simultaneously, the median test and Kruskal–Wallis test were utilized. Finally, the sign test and Wilcoxon test were employed. All calculations were performed using the STATISTICA package for statistical analysis, version 13.3.

## 3. Results

**Figure 1:**
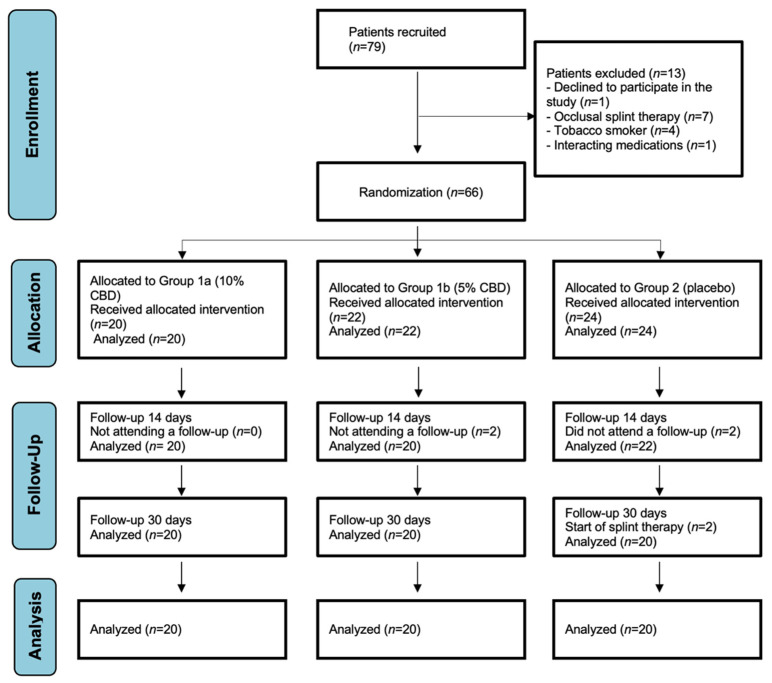
Consolidated Standards of Reporting Trials (CONSORT) flow chart of the study participants.

Out of seventy-nine patients initially assessed for eligibility, thirteen were excluded either due to not meeting the eligibility criteria or declining to participate in the study. The remaining 66 patients were randomized into the study groups. However, due to dropouts, a total of 40 patients in the intervention groups (1a and 1b) and 20 in the control group (2) completed the scheduled treatment. The number of participants is depicted in the flow diagram presented in Figure 1.

### 3.1. Descriptive Statistics

The basic demographic and clinical characteristics of the patients studied in all three groups are shown in Table 2. The mean age in Group 1a (CBD 10%) was 24.2 years, while in Group 1b (5% CBD), the mean age was 24 years. In the control Group 2, the mean age was 23.4 years. Among the patients, 24 were males (40%), and 36 were females (60%). In addition, Table 2 presents the descriptive statistics (medians and interquartile ranges) for the VAS variables at the beginning of therapy (Day-0 VAS), as well as after 14 days (Day-14 VAS) and after 30 days (Day-30 VAS). Positional mean measures were used (instead of the arithmetic mean and standard deviation) due to the ordinal nature of these variables. As can be seen, the median value of the VAS variable after 30 days is significantly lower than the value of this variable at the beginning of therapy. However, in the case of patients receiving the placebo, such a difference is not observed (6.0 vs. 5.5).

Assuming a typical confidence level of 0.95 (i.e., 95%), the confidence intervals were determined for the mean values and standard deviations of the continuous-type variables characterizing the level of significant indicators at the end of the therapy: BRK2, EMG1-L, EMG3-L, and EMG3-R. The results for both the mean values and standard deviations (SDs) are presented in Table 3.

Note, that in the case of the confidence intervals for the average value, the ranges are clearly smaller for patients receiving the 10% CBD and 5% CBD gel compared to the patients receiving the placebo. This results in a more precise estimation of the mean value for the first two groups of patients. The narrower confidence intervals for the mean values for the 10% CBD and 5% CBD gels are associated with smaller standard deviations for the respective variables for these two groups of patients. In other words, the results for the patients receiving the placebo are significantly more diverse than for the patients receiving the 10% CBD gel. This proves the effectiveness of the applied therapy, e.g., the EMG assessments for the patients receiving the drug are clearly more like to each other (health improvement is noticed by most of them).

### 3.2. Normality Tests

Table 4 presents the results of the normality tests for the studied variables. The Shapiro–Wilk test was used, and the *p*-values of this test are given in the table.

The results of the ANOVA test are presented in Table 5, where the values of the studied variables are compared for the three groups of patients. The table presents the values of the F test statistic, as well as *p*-values. As can be seen, for the variables VAS2, VAS3, BRK1, as well as EMG3-R and EMG3-L, the *p*-values are very small. This proves the existence of significant differences between the values of the examined features in individual groups of patients. Post hoc tests were then performed for these four variables, namely NIR, Scheffe, and Duncan tests to determine which groups had significant differences. The results of the post hoc tests for the individual variables are presented in Table 6, Table 7 and Table 8. Note that the tests performed indicate the occurrence of very significant differences in the level of tested characteristics between the groups of patients receiving the 10% CBD gel and the placebo (all three post hoc tests clearly indicate this). This, therefore, confirms the hypothesis about the effectiveness of 10% CBD therapy. In the case of the 5% CBD treatment compared to the placebo, differences are also visible, but not for all variables.

### 3.3. Distribution Compatibility Tests for Selected Pairs of Independent Variables

The null hypothesis tested was the hypothesis on the identical probability distributions of selected variables. Therefore, the rejection of the null hypothesis indicates significant differences in the distributions. The results obtained separately for selected variables and groups of patients are presented in Table 9. They contain the *p*-values of the relevant tests (Wald–Wolfowitz test, Kolmogorov–Smirnov test, Mann–Whitney U test).

Note that no significant differences were observed in the probability distributions of selected variables, when comparing the results for the 10% CBD and 5% CBD gel. Significant differences in the distributions were observed when comparing patients treated with the 10% CBD or the 5% CBD gel with those treated with the placebo. The obtained results confirm the significant positive impact of CBD therapy on the assessment of the patients’ complaints, expressed by the VAS, BRK, and EMG indicators.

Considering all three groups of patients at the same time, the Kruskal–Wallis and median tests were used to compare the probability distributions of selected variables.

The results are presented in Table 10. Let us note that only in the case of the BRK2 variable (bruxism index after the end of therapy), the null hypothesis on the consistency of the probability distributions of this feature in all three groups of patients could not be rejected.

### 3.4. Test for Probability Distributions of Dependent Samples

Assuming a significance level of 0.05 for selected pairs of dependent (related) variables, i.e., the values of indicators obtained for the same patients at the beginning and at the end of therapy, the following tests were applied: the Wilcoxon test, and the sign test.

The results are presented in Table 6 for patients receiving the 10% CBD gel, the 5% CBD gel, and the placebo. The table contains the *p*-values from the tests. The null hypothesis on the identity of the distributions is tested. 

The results contained in Table 11 for the patients obtaining the 10% CBD gel and the 5% CBD gel indicate significant differences in the probability distributions of the VAS, BRK, EMG-L, and EMG-R indices at the beginning and at the end of therapy in patients treated with CBD (the only exception is the EMG1-L vs. EMG3-L test). This clearly proves the effectiveness of the therapy used, both in the case of the 10% CBD and 5% CBD gels. For patients receiving the placebo, none of the tested null hypotheses could be rejected, which means that the probability distributions of the selected variables do not differ significantly at the beginning and at the end of the study period.

## 4. Discussion

In this randomized study, the authors investigated the impact of intraoral CBD use on muscle tension, pain sensations, and the level of bruxism. The findings revealed a significant correlation between intraoral CBD application and a statistically significant reduction in muscle tension, the intensity of sleep bruxism, and pain. Patients administered with a higher concentration of CBD formulation (10%) demonstrated notable improvements in pain sensations, muscle tension, and bruxism intensity, compared to those receiving the lower CBD concentration (5%). Interestingly, individuals receiving the placebo showed minimal changes to those parameters. None of the patients in the study groups reported experiencing side effects from the products. These results align with numerous studies confirming the anti-inflammatory and myorelaxant properties of CBD [30,31]. However, to the authors’ knowledge, this study marks the first investigation into the effects of intraoral application of CBD on pain and muscle tension reduction. A similar study, conducted by Nitecka-Buchta et al. (2019), explored the effects of CBD through extraoral application. In the following double-blind trial, a CBD formulation applied over masseter muscles led to reduced muscle activity and improved the condition of the masticatory muscles in patients with myofascial pain. Notably, Nitecka-Buchta et al. demonstrated that a 14-day extraoral application of a 1.46% CBD formulation resulted in a 70.2% reduction in the patients’ pain and a decrease in the sEMG activity by 11 to 12.6% [32]. Despite the varying concentrations of CBD, it can be concluded that extraoral application yields more favorable results in terms of the patients’ pain sensations. However, intraoral application demonstrates a greater reduction in the sEMG results and a reduction in sleep bruxism activity. In 2015, Hoggart et al. conducted a multicenter study to explore the effects of intraoral CBD application in conjunction with THC for alleviating neuropathic pain [33]. The outcomes of this investigation demonstrated that THC/CBD spray presents an enduringly effective approach to managing neuropathic pain. In addition, patients who continued to use the THC/CBD spray throughout the study did not increase their daily dose, nor did they seek to augment their use of other pain medications over time [33].

In 2014, Serpell et al. also reported similar results regarding the intraoral use of THC/CBD for neuropathic pain [34]. Additionally, in 2021, Vivanco-Estela et al. conducted an animal study involving rats, utilizing CBD injections into the masseter muscle to alleviate allodynia and hyperalgesia. Their investigation unveiled distinct gender-related variations in the bodily responses, with males exhibiting a more substantial decrease in hyperalgesia at equivalent doses [35]. The authors of this study observed different results, with pain reduction in men being 54% in Group 1a and 40.5% in Group 1b after 30 days, while in women, the reductions were 60.6% and 41%, respectively. The study had several strengths. A double-blinded, randomized, controlled design was deliberately selected, ensuring a robust methodology for evaluating the effects of the study intervention. The use of placebos in the study enabled the exclusion of other factors affecting the results. Each participant underwent comprehensive sleep bruxism confirmation utilizing a Bruxoff device, which achieves excellent diagnostic accuracy with respect to polysomnography (PSG) for the diagnosis of SB [28,36,37,38]. The study employed objective and reproducible methods of assessing muscle tone with the Neurobit Optima 4.0 apparatus. The reliability and reproducibility of the results was also attributed to the strict inclusion and exclusion criteria applied in this study, ensuring the homogeneity of the study group. The design of the three-arm study allowed for not only determining the relationship between CBD and TMDs, but also for exploring the correlation between higher CBD concentrations and lower muscle tension, lower pain sensation on the VAS scale, and lower sleep bruxism activity. However, the study also has some limitations related to its design. To ensure a homogeneous study population conducive to the identification of TMDs and to maintain reproducibility across the study and control groups, strict inclusion and exclusion criteria were applied. Unfortunately, this approach led to the exclusion of almost 17% of the initially screened patients from study eligibility, making it more challenging to directly extrapolate our results to actual clinical cases. Furthermore, it is worth noting that despite providing patients with training in product use, absolute confidence in the proper use of the product cannot be guaranteed, given that the administration was conducted autonomously in the participants’ households. To mitigate the potential impact of inconsistent adherence, the authors of the study conducted careful verification at each of the two follow-up visits to ensure the accurate use of the product. Additionally, the design included the establishment of separate daily doses, effectively preventing differences in dosing among the patients. Furthermore, we do not fully understand the extent of the placebo effect. Therefore, it is challenging to ascertain how the formulation influenced the control group, considering this as a basis for the emergence of TMDs. Moreover, another limitation pertains to the relatively limited diversity within the study group, owing to the single-center nature of our investigation. Consequently, the generalizability of our findings to broader patient demographics may be somewhat constrained. Thus, it is recommended that future studies adopt a multicenter approach to enhance patient diversity and bolster the external validity of the results. 

## 5. Conclusions

The intraoral use of CBD formulations in patients with TMDs has been proven to be a successful treatment for reducing pain, muscle tension, and bruxing activity in individuals with sleep bruxism and muscle-related TMDs. Specifically, a concentration of 10% CBD has demonstrated superior results compared to 5% CBD. Further investigation into the efficacy of CBD alone and in combination with occlusal splints is warranted. Additionally, exploring alternative concentrations of CBD to determine the optimal dosage is advisable.

## Figures and Tables

**Table 1:** The inclusion and exclusion criteria for patient qualification in the study.

Inclusion Criteria	Exclusion Criteria
Patients who agreed to participate in the study. Patient’s age within ≥18 and ≤60 years. Good general health. TMD positive as per the Polish version of the Research Diagnostic Criteria for Temporomandibular Disorders (RDC/TMDs) for group II.1.A. 1, 2, and 3. Presence of all teeth (except for third molars).	Cannabis formulation/placebo formulation allergy, hypersensitivity to substances to be used in the study. Wounds within oral mucosa. Addiction to cannabis. Tobacco smokers. Patients being treated with analgesic drugs and/or drugs that affect muscle function. Patients being treated with oral appliances. Fixed or removable dental prosthesis. Disease or autoimmune disorder associated with generalized muscular tension. Patients undergoing orthodontic treatment. Patients treated by a neurologist for neurological conditions. Patients with psychiatric conditions. Patients having undertaken radiotherapy, patients with an active neoplastic process. Patients with odontogenic pain. Pregnant and breastfeeding patients. Use of medications that interact with CBD. Use of any products containing cannabis.

**Table 2:** Baseline characteristics of the study participants.

	Group 1a (10% CBD) (*n* = 20)	Group 1b (5% CBD) (*n* = 20)	Group 2 (Placebo) (*n* = 20)	Total
**Age, years**	24.2	24	23.4	23.87
**Sex (%)**				
**Female**	13 (65%)	11 (55%)	12 (60%)	36 (60%)
**Male**	7 (35%)	9 (45%)	8 (40%)	24 (40%)
**Day-0 VAS ^a^**	6.0 (2.0)	6.0 (2.0)	6.0 (3.5)	6.0 (3.0)
**Day-14 VAS ^a^**	4.0 (2.0)	4.5 (3.0)	5.0 (2.5)	4.0 (2.0)
**Day-30 VAS ^a^**	2.0 (3.0)	3.5 (2.0)	5.5 (3.0)	4.0 (3.0)

^a^ Data are presented as medians (interquartile range).

**Table 3:** Confidence intervals for mean values and standard deviations for all groups of patients.

	Variable	CBD 10%	CBD 5%	Placebo
**Mean**	BRK2	(1.68, 3.20)	(1.17, 3.08)	(2.22, 3.95)
	EMG3-L	(0.08, 0.12)	(0.07, 0.16)	(0.17, 0.24)
	EMG3-R	(0.08, 0.14)	(0.06, 0.14)	(0.16, 0.27)
**SD**	BRK2	(1.00, 2.16)	(1.20, 2.67)	(1.40, 2.69)
	EMG3-L	(0.03, 0.07)	(0.06, 0.13)	(0.05, 0.10)
	EMG3-R	(0.04, 0.08)	(0.05, 0.12)	(0.09, 0.17)

**Table 4:** ANOVA and post hoc tests.

Variable	CBD 10%	CBD 5%	Placebo
VAS1	0.52468	0.36842	0.37981
VAS2	0.22472	0.06656	0.14005
VAS3	0.09676	0.09414	0.70747
BRK1	0.00187	0.00405	0.22997
BRK2	0.01062	0.03192	0.03301
EMG1-R	0.21375	0.12101	0.49225
EMG1-L	0.54563	0.25101	0.12990
EMG2-R	0.76597	0.18501	0.08086
EMG2-L	0.53321	0.46466	0.09049
EMG3-R	0.68388	0.01072	0.16111
EMG3-L	0.42479	0.26468	0.08942

**Table 5:** Results of the ANOVA tests (test values statistics and *p*-values).

Variable	F Test Statistic	*p*-Value
VAS1	0.14746	0.863302
VAS2	2.92455	0.063735
VAS3	17.72653	0.000002
BRK1	2.80636	0.070799
BRK2	1.49630	0.234654
EMG1-R	0.10407	0.901373
EMG1-L	0.13796	0.871494
EMG2-R	0.82647	0.443984
EMG2-L	1.34948	0.269450
EMG3-R	8.98313	0.000509
EMG3-L	13.18464	0.000030

**Table 6:** The *p*-values of the post hoc tests for the VAS3 variable.

	NIR	Scheffe	Duncan
Placebo vs. 5% CBD	0.000416	0.001845	0.000621
Placebo vs. 10% CBD	0.000001	0.000004	0.000065
5% CBD vs. 10% CBD	0.092035	0.238203	0.077622

**Table 7:** The *p*-values of the post hoc tests for the EMG3-R variable.

	NIR	Scheffe	Duncan
Placebo vs. 5% CBD	0.000601	0.002607	0.001068
Placebo vs. 10% CBD	0.001216	0.005047	0.001857
5% CBD vs. 10% CBD	0.776340	0.960026	0.765750

**Table 8:** The *p*-values of the post hoc tests for the EMG3-L variable.

	NIR	Scheffe	Duncan
Placebo vs. 5% CBD	0.000264	0.001199	0.000428
Placebo vs. 10% CBD	0.000030	0.000149	0.000132
5% CBD vs. 10% CBD	0.582507	0.858344	0.564200

**Table 9:** Test results for comparison between the 10% CBD vs. 5% CBD, 10% CBD vs. placebo, and 5% CBD vs. placebo.

	Variable	Wald–Wolfowitz Test	Kolmogorov–Smirnov Test	Mann–Whitney U Test
**10% CBD vs. 5% CBD**	VAS3	0.340484	>0.10	0.063618
	BRK2	0.854949	>0.10	0.419433
	EMG3-L	0.854949	>0.10	0.930443
	EMG3-R	0.574471	>0.10	0.406976
**10% CBD vs. Placebo**	VAS3	0.004315	>0.100	0.000048
	BRK2	0.452590	<0.005	0.368121
	EMG3-L	0.000378	<0.001	0.000262
	EMG3-R	0.960064	<0.025	0.004148
**5% CBD vs. Placebo**	VAS3	0.107652	<0.05	0.002200
	BRK2	0.848901	>0.10	0.093026
	EMG3-L	0.048987	<0.05	0.002936
	EMG3-R	0.048987	<0.05	0.001737

**Table 10:** Results of the Kruskal–Wallis and median tests.

Compared Variables	Kruskal–Wallis Test	Median Test
**VAS3**	0.0000	0.0001
**BRK2**	0.2274	0.4849
**EMG3-L**	0.0004	0.0010
**EMG3-R**	0.0014	0.0241

**Table 11:** Wilcoxon and sign tests for patients obtaining the 10% CBD gel, the 5% CBD gel, and the placebo.

	10% CBD		5% CBD		Placebo	
Variables	Wilcoxon Test	Sign Test	Wilcoxon Test	Sign Test	Wilcoxon Test	Sign Test
VAS1 vs. VAS3	0.000655	0.000301	0.003346	0.002569	0.722108	1.000000
BRK1 vs. BRK2	0.000655	0.000301	0.000982	0.000512	0.695093	0.813664
EMG1-L vs. EMG3-L	0.013488	0.038867	0.074736	0.267258	0.073603	0.121335
EMG1-R vs. EMG3-R	0.000982	0.000512	0.001474	0.000874	0.075869	0.145610

## Data Availability

The raw data supporting the conclusions in this article will be made available by the authors on request.
